# Enterovirus D68–Associated Acute Respiratory Distress Syndrome in Adult, United States, 2014

**DOI:** 10.3201/eid2105.142033

**Published:** 2015-05

**Authors:** John J. Farrell, Ossama Ikladios, Kristine M. Wylie, Lindsay M. O’Rourke, Kristin S. Lowery, Jenna S. Cromwell, Todd N. Wylie, Elsa L. Vazquez Melendez, Yves Makhoul, Rangarajan Sampath, Robert A. Bonomo, Gregory A. Storch

**Affiliations:** University of Illinois College of Medicine, Peoria, Illinois, USA (J.J. Farrell, E.L. Vasquez Melendez);; Saint Francis Medical Center, Peoria (O. Ikladios, L.M. O’Rourke);; Washington University School of Medicine, St Louis, Missouri, USA (K.M. Wylie, T.N. Wylie, G.A. Storch);; Ibis Biosciences, Carlsbad, California, USA (K.S. Lowery, J.S. Cromwell, R. Sampath);; Pekin Hospital, Pekin, Illinois, USA (Y. Makhoul);; Louis Stokes Cleveland Department of Veterans Affairs Medical Center, Cleveland, Ohio, USA (R.A. Bonomo);; Case Western Reserve University, Cleveland (R.A. Bonomo)

**Keywords:** acute respiratory distress syndrome, ARDS, enterovirus, EV-D68, molecular microbiology, culture-negative infections, viruses, adult, United States, respiratory infections

**To the Editor:** Each year, nonpolio enteroviruses cause 10–15 million infections in the United States (*1*). Enterovirus D68 (EV-D68) is an uncommon strain of nonpolio enterovirus that emerged in Illinois and Missouri in August 2014 in association with severe respiratory infections in children and spread across the United States (*2*). On August 23, 2014, the infection control department for Comer’s Children’s Hospital at the University of Chicago initially notified the Centers for Disease Control and Prevention of an increased number of children hospitalized with unusually severe respiratory illness (*3*). From mid-August to December 4, 2014, there were 1,121 laboratory-confirmed cases of EV-D68 in the United States (*2*). Almost all EV-D68 infections have occurred in children, many of whom had a history of asthma or wheezing (*2*).

One day before the first report (August 22, 2014), a 26-year-old obese woman with an unremarkable medical history was transferred to the medical intensive care unit at Saint Francis Medical Center, a tertiary care medical center in Peoria, Illinois, USA, with severe acute respiratory distress syndrome (ARDS). The transfer was from a nearby community hospital where she had sought care 4 days earlier for influenza-like symptoms consisting of cough, wheezing, progressive shortness of breath, nausea, and vomiting. In the community hospital emergency department, she mentioned that 2 children at home had similar symptoms and that her mother had recently been hospitalized with an acute respiratory illness. Despite treatment with supplemental oxygen, nebulized albuterol, and intravenous antimicrobial drugs for community-acquired pneumonia, her condition deteriorated, and she was intubated on hospital day 2, after which the antimicrobial drug treatment was changed from intravenous ceftriaxone and azithromycin to intravenous vancomycin and piperacillin/tazobactam. Results of bronchoscopy performed on hospital day 4 were unremarkable, and bacterial cultures of alveolar lavage samples were negative. 

Her transfer to St. Francis Medical Center was prompted by persistent mechanical ventilation requirements of 100% fraction of inspired oxygen; positive end-inspiratory pressure of 12 mm/H_2_O consistent with classic ARDS (hypoxemia, indicated by a ratio of arterial oxygen partial pressure to fractional inspired oxygen <200 mm Hg); and bilateral infiltrates on chest radiograph (Figure) without evidence of left heart failure (*4*). On hospital day 8 (cumulative), a nasopharyngeal swab sample was tested by FilmArray Respiratory Panel multiplex PCR (BioFire Diagnostics, Salt Lake City, UT, USA); results were positive for rhinovirus/enterovirus. That day, intravenous methylprednisolone therapy was initiated.

**Figure:**
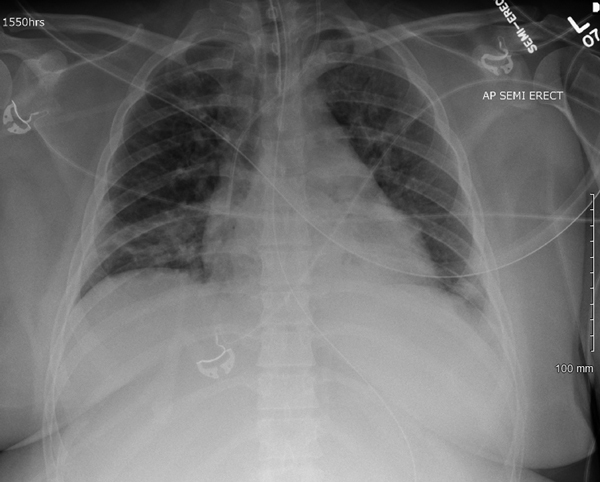
Chest radiograph obtained (with portable machine) of semirecumbent adult patient with enterovirus D68–associated acute respiratory distress syndrome on hospital day 3.

During a prolonged hospital stay, the patient required mechanical ventilation for 32 days, underwent a second bronchoscopic evaluation, required a percutaneous tracheostomy (and subsequent decannulation), and underwent endoscopic gastrostomy tube placement (and removal). She was discharged from the hospital after 55 days and ultimately recovered completely.

To determine the etiology of the clinical syndrome for the patient reported here, molecular diagnostic testing of respiratory tract clinical specimens was required. Institutional review board approval was obtained for molecular diagnostics and sequencing of the patient’s nasopharyngeal swab specimens and bronchoalveolar lavage (BAL) fluid samples. The FilmArray platform is capable of detecting enteroviral infections caused by EV-D68 but cannot differentiate between rhinoviruses and enteroviruses (*5*). Confirmation of EV-D68 requires EV-D68–specific PCR (*6*). 

A novel, research-based diagnostic modality that is capable of rapid identification of viral pathogens directly from clinical specimens is the combination of PCR and electrospray ionization mass spectrometry (ESI-MS) (*7*), which was instrumental in early recognition of the novel pandemic strain of influenza A(H1N1) virus that emerged in 2009 (*8*). For a variety of viral pathogens, PCR/ESI-MS sensitivity is 94% and specificity is 98% (*9*). In this case, PCR/ESI-MS detected a human enterovirus from the right middle lobe and left lingular segment BAL fluid samples. For the assay, 2 primer pairs were used; both confirmed the presence of human enterovirus, but only 1 matched the signatures for EV-D68. 

For confirmation, we pursued testing with EV-D68–specific PCR, which was performed by the Special Projects Laboratory of the Washington University Department of Pediatrics. This assay amplifies a segment of the viral protein 1 gene, which enables discrimination of EV-D68 from other enteroviruses and rhinoviruses (K.M. Wylie et al., unpub. data). The nasopharyngeal swab sample and the right middle lobe and lingula BAL fluid specimens were positive for EV-D68.

PCR/ESI-MS of BAL fluid followed by EV-D68–specific PCR testing of 1 nasopharyngeal swab and 2 BAL fluid samples confirmed our clinical suspicion of ARDS secondary to EV-D68 in an adult. The patient’s history of contact with sick family members and clinical signs (nonproductive cough, nausea, and vomiting) were suggestive of a viral infection. Lessons learned from the emergence of swine-origin influenza A(H1N1)pdm09 virus and recognition (in the midst of the pandemic) that younger age and obesity were risk factors for severe disease were also suggestive of a viral respiratory infection. 

We are developing a specific rapid molecular assay for EV-D68, which should help clinicians recognize when EV-D68 is present in the community. During those times, EV-D68 infection should be included in the differential diagnosis of severe respiratory infection. Documentation of EV-D68 infection may help with clinical management for individual patients and minimize unnecessary use of antimicrobial drugs within communities.

## References

[1] Centers for Disease Control and Prevention. Nonpolio enterovirus and human parechovirus surveillance—United States, 2006–2008. MMWR Morb Mortal Wkly Rep. 2010;59:1577–80 . 21150865

[2] Centers for Disease Control and Prevention. Non-polio enterovirus: enterovirus D68 in the United States, 2014 [cited 2015 Jan 8]. http://www.cdc.gov/non-polio-enterovirus/outbreaks/EV-D68-outbreaks.html

[3] Midgley CM, Jackson MA, Selvarangan R, Turabelidze G, Obringer E, Johnson D, Severe respiratory illness associated with enterovirus D68—Missouri and Illinois. MMWR Morb Mortal Wkly Rep. 2014;63:798–9 . 25211545 PMC4584696

[4] Bernard GR, Artigas A, Brigham KL. Carlet J, Falke K, Hudson L, et al. The American-European Consensus Conference on ARDS: definitions, mechanisms, relevant outcomes, and clinical trial coordination. Am J Respir Crit Care Med. 1994;149:818–24. 10.1164/ajrccm.149.3.7509706 7509706

[5] Couturier MR, Barney T, Alger G, Hymas WC, Stevenson JB, Hillyard D, Evaluation of the FilmArray® Respiratory Panel for clinical use in a large children's hospital. J Clin Lab Anal. 2013;27:148–54 . 10.1002/jcla.21576 23424157 PMC6807554

[6] Wylie KM, Wylie TN, Orvedahl A, Buller RS, Herter BN, Magrini V, Genome sequence of enterovirus D68 from St. Louis, Missouri, USA. Emerg Infect Dis. 2015;21:184–6 . 10.3201/eid2101.141605 25532062 PMC4285240

[7] Farrell JJ, Sampath R, Ecker DJ, Bonomo RA. ‘Salvage microbiology’: detection of bacteria directly from clinical specimens following initiation of antimicrobial treatment. PLoS ONE. 2013;8:e66349 . 10.1371/journal.pone.0066349 23825537 PMC3692526

[8] Metzgar D, Baynes D, Myers CA, Kammerer P, Unabia M, Faix DJ, Initial identification and characterization of an emerging zoonotic influenza virus prior to pandemic spread. J Clin Microbiol. 2010;48:4228–34. 10.1128/JCM.01336-10 20861338 PMC3020883

[9] Metzgar D, Lovari R, Ray K, Baynes D, Drapp D, Frinder M, Analytical characterization of an assay designed to detect and identify diverse agents of disseminated viral infection. J Clin Virol. 2014;59:177–83 . 10.1016/j.jcv.2013.12.005 24440177

